# Plant-Based Oil-in-Water Food Emulsions: Exploring the Influence of Different Formulations on Their Physicochemical Properties

**DOI:** 10.3390/foods13040513

**Published:** 2024-02-07

**Authors:** Carolina Quezada, Matías Urra, Camila Mella, Rommy N. Zúñiga, Elizabeth Troncoso

**Affiliations:** 1 Doctoral Program in Materials Science and Process Engineering, Universidad Tecnológica Metropolitana, Las Palmeras 3360, Ñuñoa, Santiago 7800003, Chile; 2 School of Chemistry, Universidad Tecnológica Metropolitana, Las Palmeras 3360, Ñuñoa, Santiago 7800003, Chile; murra@utem.cl; 3 Department of Biotechnology, Universidad Tecnológica Metropolitana, Las Palmeras 3360, Ñuñoa, Santiago 7800003, Chile; c.mellac@utem.cl (C.M.); rommy.zuniga@utem.cl (R.N.Z.); 4 Universitary Institute for Research and Technology Development (UIRTD), Universidad Tecnológica Metropolitana, Ignacio Valdivieso 2409, San Joaquín, Santiago 8940577, Chile; 5 Department of Chemistry, Universidad Tecnológica Metropolitana, Las Palmeras 3360, Ñuñoa, Santiago 7800003, Chile

**Keywords:** O/W emulsion, plant-based emulsifiers, soy lecithin, Quillaja saponin, microstructure, rheology, physical stability

## Abstract

The global focus on incorporating natural ingredients into the diet for health improvement encompasses ω-3 polyunsaturated fatty acids (PUFAs) derived from plant sources, such as flaxseed oil. ω-3 PUFAs are susceptible to oxidation, but oil-in-water (O/W) emulsions can serve to protect PUFAs from this phenomenon. This study aimed to create O/W emulsions using flaxseed oil and either soy lecithin or Quillaja saponins, thickened with modified starch, while assessing their physical properties (oil droplet size, ζ-potential, and rheology) and physical stability. Emulsions with different oil concentrations (25% and 30% *w*/*w*) and oil-to-surfactant ratio (5:1 and 10:1) were fabricated using high-pressure homogenization (800 bar, five cycles). Moreover, emulsions were thickened with modified starch and their rheological properties were measured. The physical stability of all emulsions was assessed over a 7-day storage period using the TSI (Turbiscan Stability Index). Saponin-stabilized emulsions exhibited smaller droplet diameters (0.11–0.19 µm) compared to lecithin (0.40–1.30 µm), and an increase in surfactant concentration led to a reduction in droplet diameter. Both surfactants generated droplets with a high negative charge (−63 to −72 mV), but lecithin-stabilized emulsions showed greater negative charge, resulting in more intense electrostatic repulsion. Saponin-stabilized emulsions showed higher apparent viscosity (3.9–11.6 mPa·s) when compared to lecithin-stabilized ones (1.19–4.36 mPa·s). The addition of starch significantly increased the apparent viscosity of saponin-stabilized emulsions, rising from 11.6 mPa s to 2117 mPa s. Emulsions stabilized by saponin exhibited higher stability than those stabilized by lecithin. This study confirms that plant-based ingredients, particularly saponins and lecithin, effectively produce stable O/W emulsions with flaxseed oil, offering opportunities for creating natural ingredient-based food emulsions.

## 1. Introduction

Today’s predominant global diet is the ‘Western diet’, which is low in vegetables and fruits, with high consumption of animal products, fat, calories, sodium, and sugar [1,2]. A problem associated with this type of diet is the excess of some kinds of food and the nutritional imbalance it causes in the body [3]. Adherence to a Western dietary pattern has been associated with an increased risk of cardiovascular disease and mortality [1]. To address this situation, previous studies have investigated modifications to this type of diet to induce weight loss in obese patients by reducing their caloric intake and increasing their consumption of plant-based ingredients such as fruits, vegetables, fiber, and whole grains [4]. These ingredients provide essential nutrients and are associated with a lower risk of chronic diseases [3,5,6,7].

Lipids, particularly ω-3 PUFAs, are essential macronutrients that play a vital role in the growth and functioning of the human body, but they are present in low quantities in the Western diet [3,8]. Furthermore, in this type of diet, another issue arises concerning the ratio between ω-3 and ω-6 PUFAs in foods. In the Western diet, there is an excess of ω-6 and a low level of ω-3, resulting in a ratio of 20:1 of ω-6: ω-3, highlighting the nutritional imbalance in this type of diet mentioned earlier [9]. These ω-3 PUFAs have been shown to reduce the risk of hypertension, hypercholesterolemia, and cancer, while ω-6 PUFAs are associated with inflammation and thrombosis [9,10,11].

Fish, such as salmon, mackerel and sardines are a rich source of ω-3 PUFAs, including eicosapentaenoic and docosahexaenoic acids; however, the current consumption of essential fatty acids from these sources is insufficient due to the unsustainable capacity of fisheries to meet global demand [12,13]. An alternative way to obtain ω-3 PUFAs is by using plant-based sources rich in these nutrients. Notable examples include canola oil, walnuts, and flaxseed, with flaxseed standing out due to having the highest ω-3 PUFA content (>75% *w*/*w*), consisting mainly of α-linolenic acid [14]. However, highly polyunsaturated lipids are susceptible to oxidation caused by environmental factors such as oxygen, light, and high temperatures. This susceptibility reduces their shelf life and negatively affects the nutritional values of the oils [8,15,16,17,18].

Since most oils are not consumed in their bulk form but rather as O/W emulsions, they are typically incorporated into food creams, dressings, and desserts [18,19]. Emulsions, having a greater specific surface area than bulk lipids, are more prone to oxidation due to the presence of oil droplets in an aqueous environment, which may contain prooxidants [8,19,20]. However, the encapsulation and protection of PUFAs within an emulsifying layer can effectively repel charged metal ions and create a barrier against reactive oxygen species, safeguarding the oil droplets [18]. This barrier enhances the protection and availability of PUFAs in the gastrointestinal tract (GIT) during digestion, a crucial aspect considering the prooxidant conditions present, such as oxygen during chewing and low gastric pH. Subsequent oxidation leads to the degradation and reduction of PUFAs, diminishing their bioavailability and nutritional contribution to the human body [21,22]. The formation of a protective barrier by surfactants determines the emulsion’s stability, which must meet certain high-quality criteria. Physical stability can be evaluated by measuring the change in droplet size and observing destabilization phenomena such as creaming or phase separation [5,23,24]. However, the oxidation state and rheological properties can affect the stability of emulsions. It has been reported that using thickeners to increase the apparent viscosity of the aqueous phase induces delayed creaming between oil droplets and reduces the oxidation rate [25,26,27].

Synthetic and natural surfactants have found widespread use in the food industry, but the former is linked to health problems [28,29]. Plant-based surfactants such as phospholipids, proteins, saponins, and polysaccharides are utilized in human food [30]. Lecithins, primarily composed of phospholipids (e.g., phosphatidylcholine), can be derived from both animal and plant sources. Incorporating lecithins into diets can offer benefits to the immune system and cardiovascular health [5,31]. The structure of a phospholipid comprises two nonpolar fatty acids esterified to a glycerol backbone, with a polar phosphate head group attached to a hydrophilic tail [8]. Additionally, phospholipids form a protective bilayer structure at the emulsion’s interface, serving as a physical barrier to shield PUFAs from oxidative agents. This protective effect ensures the stability and integrity of PUFAs within the emulsion [30,32].

On the other hand, saponins are employed as foam stabilizers in beverages such as soft drinks and beers, and as food additives to enhance solubility [33]. They offer various health benefits, including anti-inflammatory, antibacterial, antiviral, anticancer, and cholesterol-lowering effects [34,35]. Saponin from Quillaja is derived from the bark of the Quillaja tree in Chile [5,36]. Saponins share a common structure characterized by a triterpene or steroid aglycone portion, connected to one or two oligo sugar groups via ether or ester bonds [37,38]. This arrangement imparts amphiphilic surfactant properties to saponins, with the hydrophilic nature attributed to the sugar moiety and the hydrophobic nature attributed to the aglycones [37]. Consequently, utilizing saponins as surfactants in PUFA-rich emulsions improves their stability and protects them against lipid oxidation due to their inherent antioxidant activity [39].

Conversely, some hydrophilic polysaccharides are not effective as emulsifiers but they can increase the viscosity of the continuous phase, thereby improving emulsion stability [24,30]. For example, modified starches act as thickening agents, reducing the mobility of oil droplets and creating an organized and viscous network that enhances emulsion stability. Additionally, incorporating a thickener in emulsions allows for the mitigation of the oxidation of PUFAs [31,40,41].

Based on the above background, we studied the impact of using plant-based ingredients in formulating flaxseed oil-in-water food emulsions on their physicochemical properties, including physical stability, microstructural characteristics, and rheological properties. We hope that the evidence found promotes the incorporation of plant-based alternatives to produce food emulsions that are healthier and more sustainable.

## 2. Materials and Methods

### 2.1. Raw Materials

Soy lecithin containing 20–25% (*w*/*w*) phosphatidylcholine, kindly provided by Blumos S.A. (Santiago, Chile), and Quillaja saponin with 8–25% (*w*/*w*) sapogenin (Saponin 84510-500G, Sigma-Aldrich, Darmstadt, Germany) were used as emulsifiers. The dispersed phase consisted of extra virgin flaxseed oil (Fontevita^®^, Valparaíso, Chile). Emulsions were thickened using Enterex^®^ Food Thickener (Victus^®^, Miami, FL, USA), which is based on modified corn starch. Monobasic potassium phosphate, dibasic potassium phosphate and sodium azide (Sigma-Aldrich, Darmstadt, Germany) were diluted in purified water obtained from a reverse osmosis purifier (RO150, Vipure, Santiago, Chile) to prepare the phosphate buffer. Hydrochloric acid 1 N and sodium hydroxide 1 N were obtained from Fischer Scientific (Pittsburgh, Hampton, NH, USA).

### 2.2. Fabrication of O/W Emulsions

O/W emulsions were prepared by dispersing the oil (dispersed phase) into the continuous phase. Two concentrations of flaxseed oil (25% and 30% *w*/*w*) were utilized for fabricating 300 g of emulsion. The continuous phase was prepared by dissolving soy lecithin or Quillaja saponin into a volume of 5 mM phosphate buffer (pH 7.0). The mixture was stirred (MS-H280-Pro, DLAB, Beijing, China) at 1000 rpm for 2 h and then stored in an incubator (BJPX-B200II, Biobase, Jinan, China) at 3 °C for 12 h before usage. The emulsion formulation was adjusted to achieve two oil:surfactant ratios (5:1 and 10:1).

Pre-emulsions were fabricated at 25 °C by blending the volume of the dispersed and continuous phases using a rotor-stator homogenizer (Model PT 2500, Kinematika Polytron™, Malters, Switzerland) at a speed of 12,000 rpm. The pre-emulsification time was 5 min for emulsions stabilized by soy lecithin and 45 s when utilizing Quillaja saponin. In the latter case, the reduced time aimed to prevent excessive foaming. Following this, the pH of each pre-emulsion was adjusted to 7.0 and, subsequently, the samples underwent a second stage of high-pressure homogenization (Panda Plus 2000, GEA, Milan, Italy) at 800 bar and 5 cycles. To assess the physical stability of emulsions, the samples were stored for 7 days at 37 °C (Venticell 111-Eco line, Brno, Czech Republic) simulating human body temperature.

### 2.3. Fabrication of O/W Emulsions with Thickener

O/W emulsions underwent thickening by incorporating the mass of Enterex^®^ Food Thickener at a concentration of 4.0% (*w*/*w*) based on the total emulsion mass (100 g). The thickener was added in portions of 1/3 (4.0 g) and mixed for 5 min within the emulsion (96.0 g) using a spatula. Subsequently, the sample was allowed to rest for 10 min. This procedure was iterated three times until the thickener was completely integrated into the emulsion. Any residual undissolved thickener was further incorporated into the mixture through continued manual mixing with a spatula for an additional 5 min. This mixing sequence was repeated at 60, 90, 120, and 150 min from the start of the thickening stage. Finally, the thickened emulsions were stored at 37 °C until subsequent analysis.

### 2.4. Determination of Critical Micelle Concentration (CMC) between Surfactant Dispersions and Flaxseed Oil

The CMC is defined as the saturation point at which an aqueous solution containing surfactant molecules reaches the lowest value of stable surface tension and, above this point, the excess surfactants aggregate together to form micelles [42]. By this definition, CMC is a key micellar quantity with which to study the self-aggregation of amphiphilic molecules in solution [43]. To determine the CMC for the two surfactants under investigation, their interfacial tension was measured at different concentrations (0.01–0.30 g/L for Quillaja saponins and 0.2–0.8 g/L for soy lecithin) using the pendant drop method. In this method, a drop of surfactant dispersion forms at the tip of a steel needle inside a cuvette containing 3.0 g of flaxseed oil. The measurements were conducted in a temperature-controlled chamber at 25 °C for 60 min. Before measurements, the flaxseed oil was purified by mixing it with resin (Florisil^®^ 60–100 mesh, 46385, Sigma Aldrich, Darmstadt, Germany) in a resin:oil ratio of 1:10 to eliminate any surfactant impurities. The mixture was agitated for 4 h and then centrifuged (320 R, Hettich, Hemelingen, Germany) at 3500 rpm and a gravity force of 1160 g for 30 min. Subsequently, the oil was filtered using a 0.45 µm syringe filter (Finetech, Taichung, Taiwan) and stored at 5 °C until use.

To measure the interfacial tension (γ) (mN/m), an optical tensiometer (Ramé-Hart, model 250-F4, Succasunna, NJ, USA) was employed. This instrument utilizes a camera to detect changes in droplet shape as the surfactant in the medium is adsorbed. The DROPimage Advanced software (Ramé-Hart Inc., Succasunna, NJ, USA) then fits a polynomial equation to the droplet shape to calculate the interfacial tension according to:

(1) $$\gamma =\frac{∆\rho \;g{\;R}_{0}^{2}}{\beta }$$

where Δρ is the density difference between the fluids of both phases (kg/m^3^), g is the acceleration due to gravity (m/s^2^), R_0_ is the initial droplet radius (m), and β is a dimensionless shape parameter. The droplet volume for each experiment was determined by establishing the Worthington number (Wo), quantifying the effect of the droplet volume on the interfacial tension as follows:

(2) $$\mathrm{Wo}=\frac{∆\rho \;g\;V_{d}}{\pi \gamma {\;D}_{n}}$$

where V_d_ is the droplet volume (m^3^) and D_n_ is the needle diameter (m). Reliable measurements of interfacial tension are assumed when the Wo number falls within the range of 0.9–1.0 [44,45].

### 2.5. Determination of Physicochemical Properties of O/W Emulsions

#### 2.5.1. Microstructure and Particle Size Distribution

The emulsion microstructure was characterized using an optical microscope (model CKX53, Olympus, Tokio, Japan) equipped with a 40× objective lens. Before visualization, samples were diluted with phosphate buffer in a 1:10 ratio (sample:buffer). A small volume of the dilution was placed on a microscope slide and covered with a coverslip. The obtained micrographs were processed and analyzed using the microscope image analysis software LCmicro 2.2, Olympus Soft Imaging Solutions GmbH (Olympus, Tokio, Japan).

To measure the oil droplet size distribution, a static light scattering particle size analyzer (Mastersizer 3000, Malvern Instruments, Worcestershire, UK) was employed. This instrument determines droplet size by analyzing angular light scattering patterns. The refractive indices of the dispersed and continuous phases were experimentally determined as 1.46 and 1.33, respectively (RSD Refractometer, Zhejiang, China), with an absorption index of 0.001 [46]. For these measurements, emulsion samples were diluted by adding a few drops to 700 mL of 5 mM phosphate buffer with a darkening close to 0.2%, adjusted to the same pH as the sample. The degree of oil droplet size dispersion in the emulsions was assessed using the polydispersity index (PDI), calculated as follows:

(3) $$\mathrm{PDI}=\frac{d_{90}-d_{10}}{d_{50}}$$

The values of d_50_, d_90_, and d_10_ represent particle sizes when the cumulative volume of particles accounts for 50%, 90%, and 10% of the total volume, respectively [47]. A higher PDI value indicates a wider size distribution.

For the preparation of emulsions stabilized by soy lecithin, there is a possibility of forming micelles or liposomes when the emulsions have a higher concentration of emulsifier [21]. This phenomenon was analyzed by preparing dilutions of soy lecithin in 5 mM phosphate buffer (300 g). Four surfactant concentrations (2.5%, 3.0%, 5.0%, and 6.0% *w*/*w*) were used to fabricate the dispersions, the same concentrations used in the preparation of the O/W emulsions. The dispersion was stirred at 1000 rpm for 2 h and then stored at 3 °C for 12 h. Subsequently, it underwent two consecutive homogenization processes, similar to the O/W emulsions. First, a rotor-stator homogenizer was used at a speed of 12,000 rpm at 25 °C and pH 7.0, followed by high-pressure homogenization at 800 bars for 5 cycles. The particle size distribution of the dilution was measured using a static light scattering particle size analyzer, and a refractive index specific to soy lecithin (1.45) was employed [48].

#### 2.5.2. ζ-Potential of Emulsions

The ζ-potential of emulsion droplets was measured using a Nanosizer analyzer (model ZEN3690, Malvern Instruments, Worcestershire, UK). This instrument determined the average charge of particles present in a sample. For this analysis, 1.0 mL of the sample was introduced into a cuvette with electrodes (cell type DTS1070, Malvern, Worcestershire, UK). The analyzer conducted five measurements for each sample, with the number of runs automatically determined by the equipment based on the specific characteristics of each sample.

#### 2.5.3. Rheological Properties of Emulsions

All rheological measurements were carried out using a rotational rheometer (AntonPaar, RheolabQC, Graz, Austria). The emulsions were carefully poured into the rheometer cup and allowed to rest for 60 s before testing. The test temperature was maintained at 37 °C throughout the assay. For non-thickened emulsions, the concentric cylinder measuring system DG42 (Anton Paar, Graz, Austria) was employed, while the concentric cylinder measuring system CC27/S (Anton Paar, Austria) was used for thickened emulsions. Two rheological tests were performed: (i) constant strain rate test, where the emulsions were subjected to a fixed shear rate of 50 s^−1^ for 60 min; and (ii) flow curve test (only for non-thickened emulsions), where the shear rate was progressively increased linearly from 0.1 to 100 s^−1^ for 45 min. The experimental flow curves of the emulsions were analyzed using the Power Law model, expressed by the following equation:

(4) $$\tau =k\;\varphi^{n}$$

where τ represents the shear stress (Pa), k is the consistency coefficient (Pa·s^n^), φ is the shear rate (s^−1^), and n corresponds to the flow behavior index (dimensionless). For Newtonian emulsions, n = 1; however, n < 1 for emulsions exhibiting shear-thinning and n > 1 for those with shear-thickening behavior. The goodness of fitting between the experimental and predicted flow curves was assessed by the coefficient of determination (R^2^) and the root mean square error (RMSE).

#### 2.5.4. Physical Stability of Emulsions

The stability of both thickened and non-thickened emulsions was analyzed over a 7-days storage period using light backscattering analysis conducted with a physical stability analyzer (Turbiscan^®^ Classic MA 2000, Formulaction, Toulouse, France). This method enabled the determination of emulsion stability and the detection of any particle migration that may not be visible to the naked eye [49].

The emulsions were placed in cylindrical glass tubes, which were then fully scanned using a light source. The transmittance detector received the light passing through the sample at a 180° angle relative to the source, while the backscatter detector captured the light scattered backward by the sample at a 45° angle. The TSI parameter was employed to quantify variations in backscatter across consecutive measurements as a function of sample height, calculated as follows:

(5) $$\mathrm{TSI}=\sum_{n}\left(\frac{\sum_{h}\left|{\mathrm{scan}}_{i}\left(h\right)-{\mathrm{scan}}_{i-1}\left(h\right)\right|}{H}\right)$$

where scan_i_(h) represents the backscatter at a certain height for a specific measurement time (i) (%), scan_i−1_(h) represents the backscatter at the same height for the previous measurement time (i − 1) (%), and H is the height of the sample (mm). TSI is determined by summing up all variations in the emulsion based on droplet size and spatial concentration. A higher TSI indicates greater instability, with lower TSI values indicating more stable emulsions due to minimal variations in transmitted light or retrodispersion (ΔBS). These variations signify a reduced occurrence of phenomena like coalescence, flocculation, sedimentation, and creaming [50]. Emulsion stability is further assessed through the ΔBS signal, which is measured along the height of the samples to define the creaminess profile. A stable emulsion shows no perceptible variation in the ΔBS signal during storage. Gravitational destabilization is identified by a negative peak in the lower part and a positive peak in the upper region. Conversely, an increase in the entire signal suggests destabilization through flocculation, coalescence, or Ostwald ripening [51,52]. All emulsion samples were stored in glass test tubes with lids at 37 °C for 7 days within a laboratory oven (Venticell 111-Eco line, Brno, Czech Republic). Daily images of the samples were captured using a digital camera (DSC RX100 V, Sony, Tokio, Japan) to capture three photos of each sample per day (n = 3).

### 2.6. Statistical Analysis of Data

All tests and analyses were conducted in triplicate using three independent samples. The results were reported as mean values ± standard deviations (SD). The datasets obtained were analyzed utilizing the statistical analysis software Statgraphics Centurion 19 (Statgraphics Technologies Inc., The Plains, VA, USA). To ascertain significant differences among the results (*p* < 0.05), analysis of variance (ANOVA) was performed, followed by Tukey’s post-hoc test.

## 3. Results and Discussion

### 3.1. Critical Micellar Concentration and Interfacial Tension

**Figure 1:**
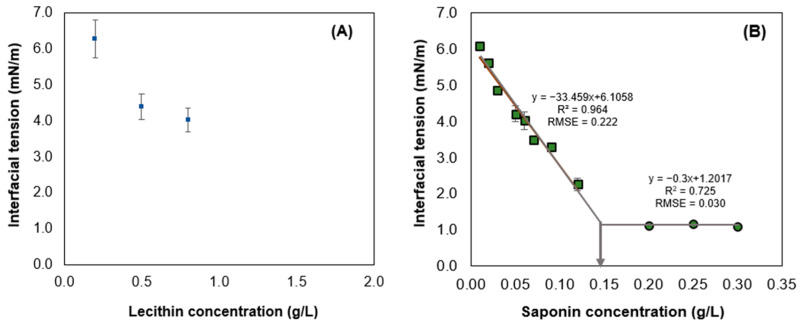
Effect of soy lecithin (**A**) and Quillaja saponin (**B**) concentrations on the equilibrium interfacial tension at the interface of 5 mM phosphate buffer aqueous solution and flaxseed oil.

The interfacial tension of surfactants plays a crucial role in understanding their ability to form stable O/W emulsions. The emulsifying efficiency is directly related to the reduction of interfacial tension between the oil–water interface [53]. Figure 1 display graphs illustrating the determination of the CMC for both soy lecithin and Quillaja saponin at different concentrations, using flaxseed oil. Both surfactants exhibited decreasing interfacial tension with increasing concentration, indicating the adsorption of these surfactants at the oil–water interface.

The CMC is defined as the concentration at which the surfactants in the aqueous phase begin to organize themselves specifically around the oil droplets, facilitating the formation and stability of the emulsion [54]. Particularly for soy lecithin, the CMC is the concentration at which the phospholipids present in lecithin begin to aggregate in the oil, a result of the hydrophobic effect that forces nonpolar segments to move away from water and come together [55,56]. Furthermore, being a low molecular weight surfactant, soy lecithin demonstrates rapid diffusion and adsorption of molecules at the oil–water interface of the oil droplet, providing an alternative to high molecular weight surfactants such as soy protein isolate and whey protein isolate [57]. However, in our case, when its concentration exceeded 0.08 g/L, the dispersions became opaque, and the equipment used could not perform measurements, hindering the determination of interfacial tension and, consequently, the CMC value. Determining an estimated value for the CMC from Figure 1A is not feasible since no intersection is observed when plotting lines corresponding to the decrease in interfacial tension and the lack of variation due to saturation at the interface. A similar situation was reported by [58], where a soy lecithin concentration exceeding 0.03% *w*/*w* resulted in opaque solutions, leading to unreliable measurements.

On the other hand, for Quillaja saponin, a more pronounced decrease in interfacial tension was observed when increasing surfactant concentration, suggesting that saponin induces a more effective saturation of interfaces [59]. In other words, as the concentration of the Quillaja saponin surfactant increases, there may be greater adsorption and coverage of the oil–water interfaces, resulting in a more significant decrease in interfacial tension. This behavior suggests an increasing effectiveness of the emulsifier in reducing interfacial tension as the concentration is increased, which is essential for the formation and stability of O/W emulsions. The estimated values of the CMC, determined from the intersection between the lines formed when the interfacial tension decreases and when there is no variation due to saturation at the interface [60], showed that the CMC of Quillaja saponin was 0.15 g/L (Figure 1B). This value is consistent with values found in the literature ranging between 0.1 and 0.8 g/L at 25 °C [61,62]. Additionally, the CMC values of synthetic surfactants such as Tween 20 and Tween 80 with flaxseed oil are around 0.12 g/L and 0.01 g/L, respectively [63,64]. The use of Quillaja saponin is a reasonable alternative, at least for replacing ingredients in many commercial applications like Tween 20 to produce stable O/W emulsions. However, Tween 80 requires a higher surfactant concentration, presenting a disadvantage compared to this synthetic surfactant [65]. Saponin is also a more efficient alternative compared to some biosurfactants. An example is the production of a biosurfactant from the yeast *Candida lipolytica*, which has a CMC of 25 g/L in canola oil, an oil known for its high content of PUFAs [66]. Considering this information, one can reasonably anticipate that employing saponins at modest concentrations to create emulsions with minute oil droplets will result in stability under specific conditions.

### 3.2. Particle Size and Microstructure

**Figure 2:**
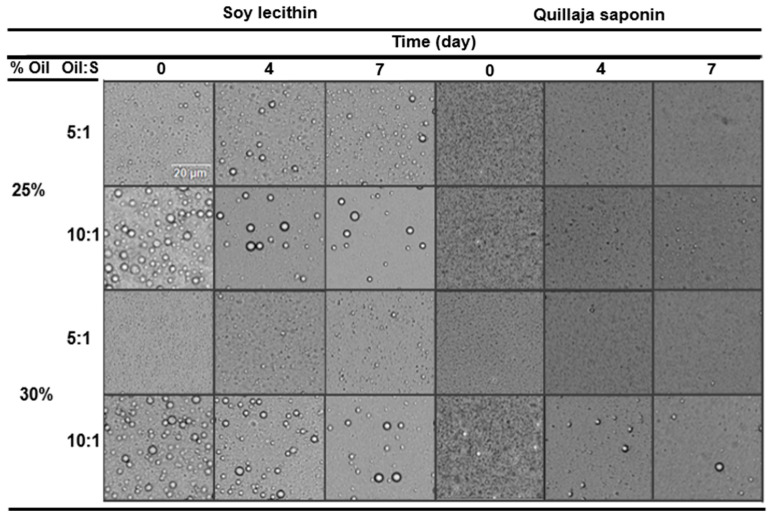
Optical micrographs of O/W emulsions stabilized by soy lecithin and Quillaja saponin. S—surfactant (scale bar = 20 μm).

Microscopy images of emulsions formulated with lecithin and saponin, without the presence of modified starch, are presented in Figure 2. The micrographs reveal that lecithin-stabilized emulsions exhibit numerous visible individual particles (Figure 2). As the surfactant concentration increases, the emulsion displays visibly smaller droplet sizes, further decreasing with an increase in oil concentration. In contrast, saponin-stabilized emulsions show no observable particles in most micrographs (Figure 2). This suggests that droplet diameters are below the microscope’s detectable limit. This observation is consistent, considering that Quillaja saponin is recognized as a highly effective surfactant for forming emulsions with small droplet sizes that are stable under a wide range of conditions [62,67,68]. Moreover, there were no significant variations in droplet size over the 7-day monitoring period, regardless of the type of emulsifying agent, oil concentration, and oil-to-surfactant ratio.

**Figure 3:**
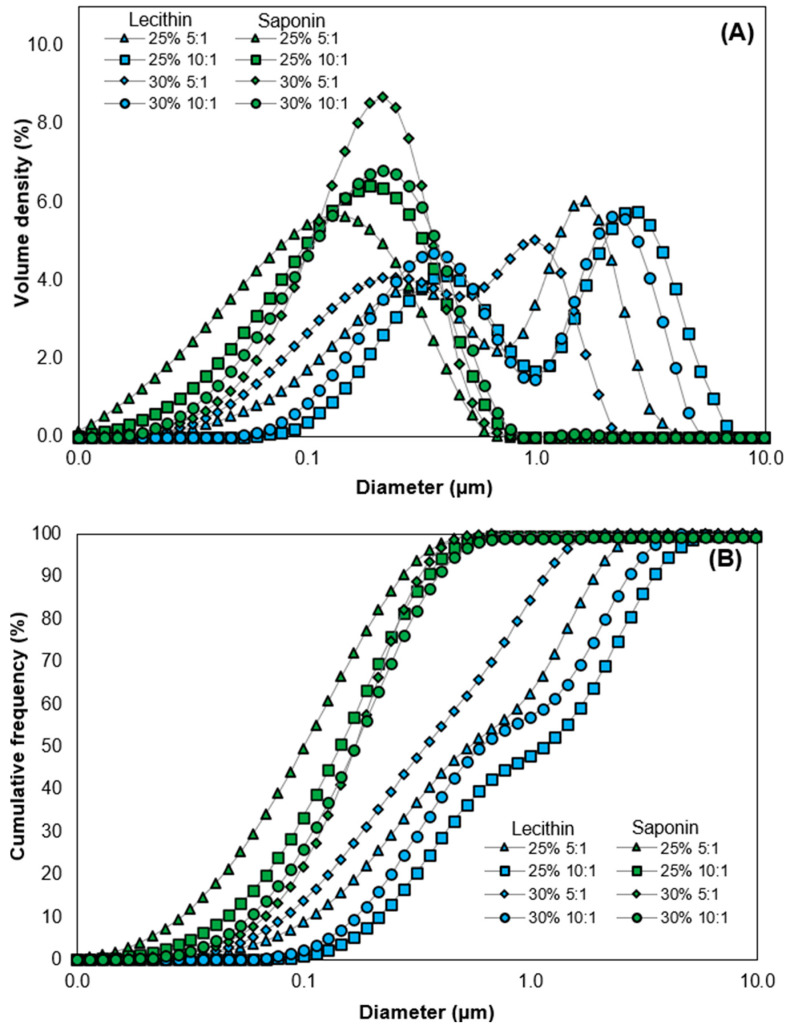
Particle size distribution (**A**) and cumulative frequency distribution (**B**) of freshly prepared O/W emulsions. Diameter axis in logarithmic scale.

The droplet distributions in the emulsions illustrated in Figure 3A were markedly different between the types of surfactants. Saponin-stabilized emulsions showed narrower and monomodal droplet size distributions compared to lecithin. Furthermore, according to the cumulative frequency distribution of droplet diameter (Figure 3B), as the concentration of both surfactants increases, there is a decrease in droplet size. Additionally, in Table 1, the values of d_50_, d_10_, d_90_, as well as the PDI for both types of emulsions are presented. Indeed, emulsions with Quillaja saponin had smaller mean diameters (d_50_: 0.11–0.19 μm) compared to lecithin (0.40–1.30 μm).

On the contrary, when soy lecithin is used, it is possible to observe a decrease in the mean droplet diameter when 30% (*w*/*w*) oil is employed in the emulsion formulation (d_50_: 0.40–0.70 μm). This behavior is attributed to the hydrophobic nature of lecithin, causing a reduction in droplet size with an increase in the oil level [68].

Regarding the PDI values (Table 1), saponin-stabilized emulsions exhibited significantly lower variability in droplet size (mean PDI: 2.0) compared with lecithin (mean PDI: 3.4). These findings align with the droplet size distributions shown in Figure 3A. Lower PDI values indicate more homogeneous droplet size distributions [69], as observed in this study. In the case of lecithin-stabilized O/W emulsions, all droplet size distributions displayed a bimodal nature. This phenomenon could be attributed to the migration of some lecithin molecules to the aqueous phase during emulsion preparation, forming micelles or vesicles, thereby increasing the effective volume fraction in the continuous phase of the emulsion [24,68]. To confirm this, dispersions containing only lecithin at the same concentrations used for emulsion fabrication were prepared using the same homogenization procedure. Subsequently, the dispersions underwent droplet size analysis. The particle size distributions indicated that the mean diameter (d_50_) of lecithin micelles was 0.07 μm, a value close to those found for the first peak in Figure 3A.

### 3.3. Measurements of ζ-Potential of O/W Emulsions Stabilized by Plant-Based Surfactants

**Figure 4:**
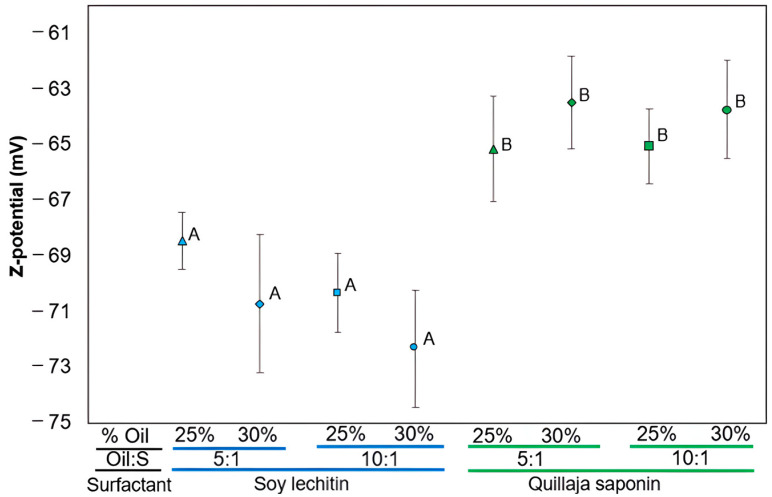
ζ-potential of O/W emulsions stabilized by soy lecithin and Quillaja saponin. Mean values were significantly different with respect to surfactants (^A,B^) (Tukey’s post-hoc, *p* < 0.05).

The importance of measuring ζ-potential lies in its relationship with the short-term and long-term stability of emulsions. Emulsions with a high zeta potential (negative or positive) are electrically stabilized, while those with a low zeta potential tend to flocculate or coagulate, resulting in poor physical stability [70]. Notably, there were significant differences in ζ-potential measurements between the two surfactants (Figure 4). ζ-potential values were lower for soy lecithin, ranging between −72 and −68 mV, and became more negative with an increase in the volume of the dispersed phase. This behavior can be attributed to the composition of soy lecithin, consisting of 13.3–24.0% *w*/*w* phosphatidylcholine (PC), with the remaining fraction mainly comprising anionic phospholipids [71]. The latter have transverse areas of alkyl chains (nonpolar) and small areas of polar head groups that show a greater affinity for the oily phase, while PC has a large area of polar head groups and smaller alkyl chains [31]. When these phospholipids are packed at the emulsion interface, the nonpolar tails orient toward the droplet surface, while the larger polar head groups face outward, in contact with the aqueous phase [72]. Therefore, the lower the proportion of PC in lecithin, the more challenging its hydration as an emulsifier, favoring the formation of emulsions with higher oil content [73]. This phospholipid arrangement results in negatively charged droplets, stable at higher oil concentrations, creating strong repulsive interactions that prevent destabilization phenomena [74]. Based on these factors, a smaller mean droplet diameter is observed in O/W emulsions with a greater negative ζ-potential, indicating the soy lecithin’s affinity for the oily phase due to the lower concentration of PC.

In the case of Quillaja saponin, emulsion droplets displayed a greater negative ζ-potential ranging from −63 to −65 mV, aligning with reported values for Quillaja saponin in O/W emulsions (−40 to −60 mV) [75,76]. This effect is attributed to the presence of carboxylic groups in the saponin structure, contributing to a negative surface potential. The droplets are primarily stabilized by electrostatic repulsion between more negatively charged droplets [53,77,78]. Additionally, negative ζ-potential values were recorded in emulsions with lower volume fractions of dispersed phase, associated with a smaller mean droplet diameter. This trend is explained by the low concentration of sapogenin, which is the hydrophobic part of the saponin structure. Most of the structure is composed of hydrophilic sugar fractions that exhibit affinity with emulsions having a higher proportion of continuous phase [79].

### 3.4. Flow Curve and Rheological Behavior of O/W Emulsions Stabilized by Plant-Based Surfactant

**Figure 5:**
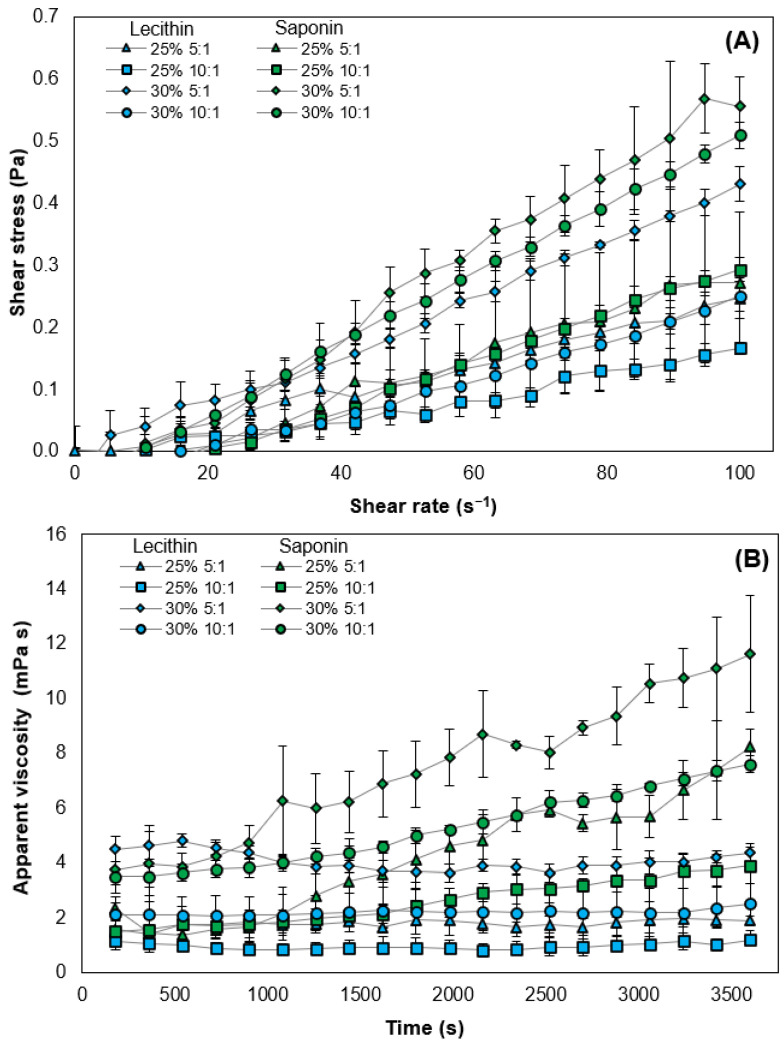
Shear stress as a function of shear rate (**A**) and apparent viscosity as a function of time (**B**) of O/W emulsions without modified starch.

The flow profiles depicting shear rate and shear stress dependence in O/W emulsions stabilized by soy lecithin and Quillaja saponin, without the presence of a thickener, are presented in Figure 5A. The curves revealed that emulsions stabilized with Quillaja saponins exhibited greater variation in shear stress as shear rate increased, compared to emulsions made with lecithin. Additionally, emulsions with a higher volume of oil phase proportion experienced a more pronounced increase in shear stress when subjected to shear rate, resulting in higher apparent viscosity. This is attributed to flaxseed oil offering greater flow resistance than water (with an oil viscosity of 32 cP and water viscosity of 0.65 cP at 40 °C), causing an increase in apparent viscosity when a higher oil percentage (*w*/*w*) in the emulsion was used [80,81].

Table 2 displays the results of fitting the flow curve to the power law model (0.02 < RMSE < 0.13; 0.93 < R^2^ < 0.99). These values revealed that most emulsions exhibited non-Newtonian behavior, specifically shear-thickening behavior. Maranzano & Wagner [82], observed a correlation between the increase in repulsive forces and the enhancement of shear-thickening behavior in dispersions [82]. Saponin emulsions with 30% oil demonstrated shear-thickening and higher apparent viscosity, attributed to greater negative ζ-potential values (between −64 and −65 mV). By presenting smaller sizes for the same oil levels, with a higher number of particles, and given their negative charge (almost at the same level as lecithin), higher repulsion and increased apparent viscosity are expected. Conversely, a greater tendency towards shear-thickening was expected from lecithin due to its high (negative) ζ-potential values. However, emulsions with 25% and 30% (*w*/*w*) oil and a higher ratio of soy lecithin mass (5:1 oil:surfactant) showed a linear relationship between shear stress and shear rate, with n values of 1.09 each, indicating Newtonian behavior. On the other hand, as K increases, there is a noticeable rise in the average fluid apparent viscosity [83]. In the case of lecithin-based emulsions, the quantity of surfactant mass plays a crucial role in this phenomenon, while in the case of saponin, it is the oil concentration that emerges as a determining factor [84].

The apparent viscosities of saponin-stabilized emulsions underwent a continuous increase during the constant application of shear over a 60 min period, as illustrated in Figure 5B. This increase was more pronounced with higher oil concentrations, indicating the presence of rheopexy in this type of emulsion. Rheopexy, comparable in time to shear-thickening, reveals the lack of necessary time for the reorganization of its structure, resulting in a significant accumulation of shear forces and an increase in apparent viscosity [85,86]. In emulsions with lecithin, the deformation rate depends solely on tension and remains constant over time [87]. Although a similar trend to saponin in ingredient proportion could have been expected, apparent viscosity is also influenced by the droplet size distribution [88]. Emulsions with saponin have smaller droplets compared to those with lecithin. As particle size decreases, the surface area for continuous phase dispersion increases, and the viscosity rises [89,90]. Conversely, emulsions with lecithin exhibit a decrease in apparent viscosity due to their bimodal distribution, allowing smaller droplets to fit between the larger ones in the streamlines during flow [68,88].

The introduction of modified starch revealed notable discrepancies in the mean apparent viscosity values, whether with or without thickener, as detailed in Table 3. This increase is noticeable for both types of surfactants, but emulsions stabilized by saponin exhibited a higher apparent viscosity (679–2137 mPa·s) than those stabilized by lecithin (370–1991 mPa·s). This disparity could be linked to the droplet size distribution, where saponin shows monomodal distributions, unlike lecithin which has bimodal distributions. In the latter, smaller droplets and micelles fit into the empty spaces of the cubic matrix of the droplet distribution, slowing down the movement of droplets compared to monomodal emulsions, resulting in lower viscosity [91,92]. However, saponins, by presenting droplet sizes smaller than those of lecithin, have lower points of interruption in the modified starch matrix in the continuous phase. This results in a more organized network, providing greater resistance to flow. Also noteworthy is the emulsion made with lecithin with 30% (*w*/*w*) oil at a ratio of 5:1 experiencing an increase from 4.36 to 1991.4 mPa s. This emulsion has a lower PDI (3.09) and a less critical degree of bimodality, as observed in lecithin emulsions. Moreover, no changes were observed in the trends of the apparent viscosity curves, except in those stabilized by saponin with 30% (*w*/*w*) oil. In this case, when thickener mass was added, there was no evident increase in the viscosity curve during the continuous application of stress, establishing a linear relationship between the deformation rate and stress (additional information in Supplementary Materials), contrary to the behavior without thickener, where a time-dependent relationship is observed in Figure 5B.

### 3.5. Stabilizing Effect of the Modified Starch in O/W Emulsions

**Figure 6:**
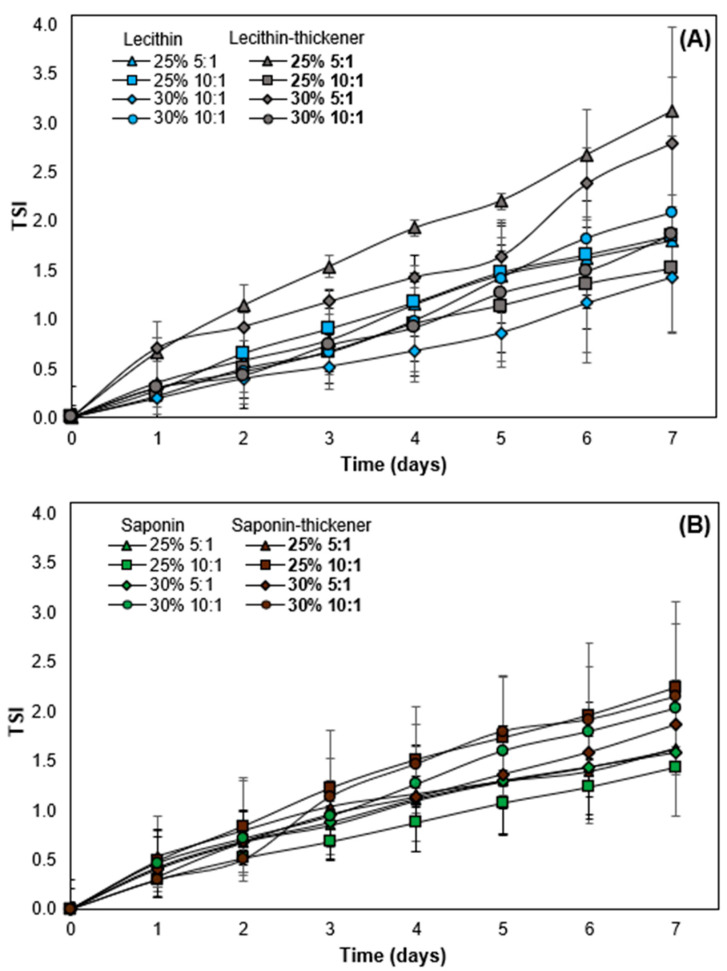
Turbiscan stability index (TSI) of O/W emulsions with and without thickener stabilized by soy lecithin (**A**) and Quillaja saponin (**B**) monitored until 7 days. Mean values were significantly different with respect to thickening addition (Tukey’s post-hoc, *p* < 0.05).

The stability of O/W emulsions with and without modified starch was evaluated by the TSI value over a 7-day monitoring period. There were similarities in TSI values between emulsions stabilized by saponin and lecithin in the absence of starch (Figure 6). Specifically, the highest stability was observed in saponin emulsions with a 10:1 ratio of oil-to-surfactant mass and 25% (*w*/*w*) oil phase, with a TSI of 1.44 after 7 days of monitoring. In contrast, emulsions with lecithin at 30% (*w*/*w*) oil and higher surfactant mass ratio showed the lowest TSI (1.43). These results may be associated with both emulsions having greater negative ζ-potential, indicating electrical stability. This suggests that repulsive interactions generated by interfacial coatings (electrostatic and steric) proved to be strong enough in both types of emulsions to counteract the Van der Waals attraction forces between droplets, preventing aggregation or flocculation, which occurs when droplets are attracted to each other [59,93]. In addition, non-thickened emulsions with saponin showed slightly lower TSI values compared to lecithin-stabilized emulsions, indicating higher physical stability for the former. This is consistent with the fact that emulsions with saponin presented smaller droplet sizes, undergoing a slower gravitational separation rate, and exhibiting a higher propensity for random dispersion attributed to the effects of Brownian motion.

**Figure 7:**
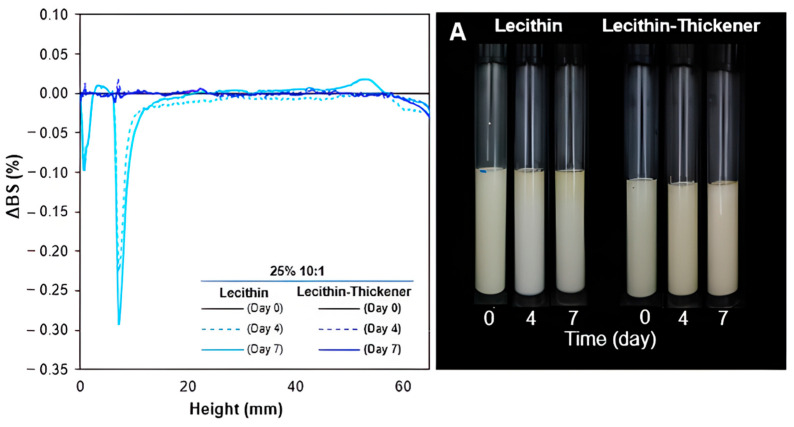

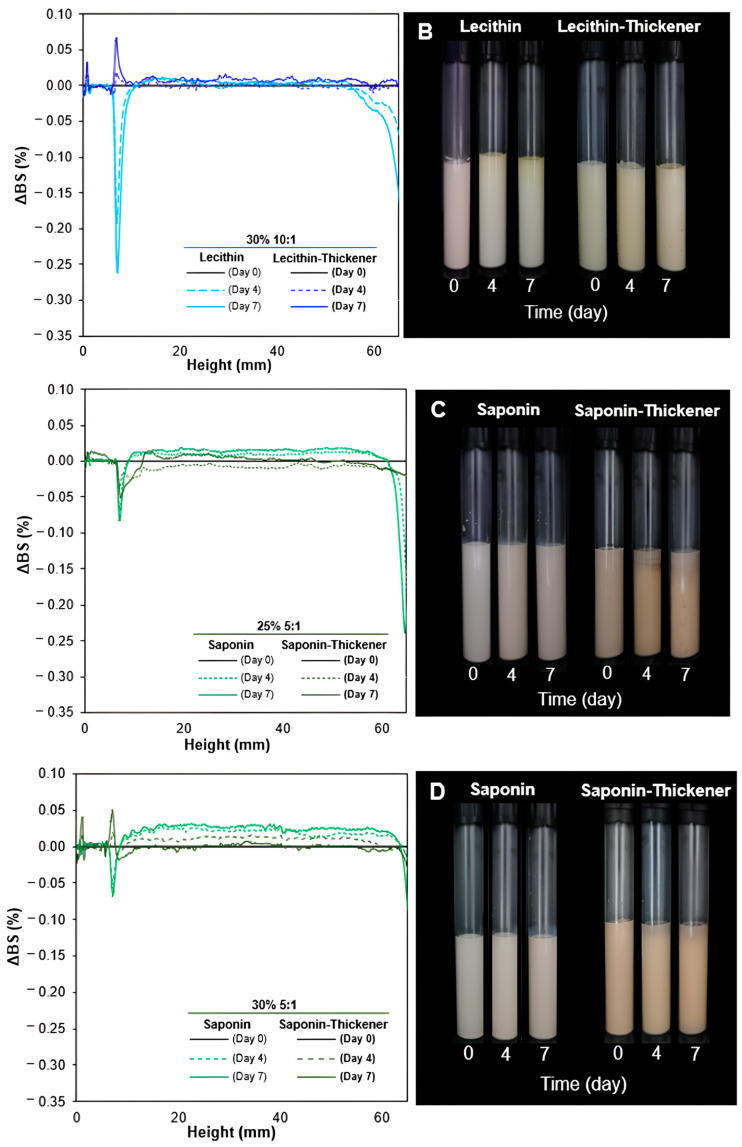
Evolution of delta backscattering (ΔBS) profiles (**left**) and a photographic record (**right**) of O/W emulsion stabilized by soy lecithin (**A**,**B**) and Quillaja saponin (**C**,**D**) with and without thickener, monitored until 7 days.

The impact of adding thickener on emulsion stability was also demonstrated, depending on the type of surfactant and the oil–surfactant ratio. This is evident in TSI values for lecithin-stabilized emulsions (Figure 6A). To complement these findings, measurements of variations in ΔBS at different heights of the O/W emulsions within the Turbiscan measurement cells were conducted (additional information in the Supplementary Materials). A decrease in TSI was observed in the case of soy lecithin surfactant when adding thickener, specifically at the 10:1 oil:surfactant ratio. Initially, the TSI was 1.9, decreasing to 1.5 with a 25% *w*/*w* oil content and from 2.1 to 1.9 when increased to 30% *w*/*w* oil content. Figure 7 compares the ΔBS values and appearance of 10:1 lecithin O/W emulsions for both oil concentrations (25% *w*/*w* and 30% *w*/*w*) without the addition of modified starch mass. The non-thickened emulsion at 25% *w*/*w* (Figure 7A) showed a decrease in ΔBS at the bottom of the glass cell (0–7 mm) and a slight increase at the top of the cell (60–65 mm). This reflects an increase in particle concentration at the top of the tube, leading to gravitational destabilization phenomena such as creaming [94]. On the other hand, the lecithin-stabilized emulsion with 30% *w*/*w* of oil and an oil:surfactant ratio of 10:1 (Figure 7B) showed destabilization through coalescence, flocculation and/or Ostwald ripening according to ΔBS measurements. Similarly, both lecithin-stabilized emulsions showed an increase in yellowness at the top of the container over 7 days (Figure 7A,B), possibly due to creaming destabilization. However, adding modified starch improved stability, correlating with a decrease in TSI in 10:1 O/W emulsions. Polysaccharides are used to enhance the viscosity of the continuous phase and reduce the destabilization rate in emulsions, thereby preventing creaming [95,96]. However, it is known that emulsion stability also depends on droplet sizes, with emulsions with large droplets being less stable than their counterparts with small droplets. The rest of the lecithin-stabilized emulsions showed minimal changes in ΔBS, indicating greater emulsion stability [97]. On the contrary, adding thickener in most saponin-stabilized emulsions increased TSI values (Figure 7B).

According to Guo et al. [98], higher viscosity contributes to increased emulsion stability. Saponin-stabilized emulsions exhibited apparent viscosity values ranging from 789 to 2133 mPa·s; however, despite the addition of a thickening agent leading to increased viscosity, no corresponding improvement in stability was observed [98]. In Figure 7C,D, a slight decrease in ΔBS was observed at the top and bottom of the container but there was an increase in the central region, indicating destabilization through coalescence or flocculation. Changes in ΔBS measurements over time could indicate an increase in droplet size, possibly due to certain combinations like surfactant-polysaccharide as a thickener that can create a robust stabilizing layer, forming an osmotic barrier due to the presence of a high concentration of polymer segments between droplets [99,100]. However, all emulsions developed a dark brown color and some creaming after 7 days. This is more pronounced in emulsions with a 5:1 oil:surfactant ratio and 30% (*w*/*w*) of oil, where the addition of modified starch leads to droplet coalescence. The choice of the appropriate polysaccharide is crucial, as it affects the rheology, stability, and interactions with the emulsion oil [101].

## 4. Conclusions

In conclusion, this comparative study of emulsions stabilized by soy lecithin and Quillaja saponin has provided crucial insights into the physical properties and stability of these formulations, particularly in the context of food emulsions made with flaxseed oil (rich in PUFAs) in water. The results highlight significant differences between the two surfactants (lecithin and saponin) shedding light on their distinctive effects on the microstructure, droplet size, electrostatic stability, and rheology of the emulsions. Lecithin-stabilized emulsions exhibited higher droplet sizes compared with those fabricated with saponin, which was confirmed by optical micrographs. The droplet size distribution was more homogeneous in saponin-stabilized emulsions, which presented monomodal distributions differing to those found for their counterparts with lecithin (with bimodal size distributions). Due to the microstructure results, the feasibility of obtaining O/W emulsions with reduced diameters was determined using low concentrations of saponin (at a ratio of 10:1 oil:surfactant). However, a drawback of using saponin is the excessive foam during emulsion manufacturing, requiring a homogenization methodology adjustment. Additionally, the evaluation of the ζ potential revealed higher electrostatic stability in lecithin-stabilized emulsions, but emulsions with saponin also showed high negative ζ-potential values, resulting in a more pronounced non-Newtonian behavior and higher apparent viscosity, especially in the presence of modified starch mass. However, the ease of micelle formation determined the apparent viscosity of lecithin O/W emulsions, and it was not possible to address this analysis entirely because quantifying an experimental CMC value proved impossible (the sample was too opaque). The addition of thickener had differentiated impacts on the emulsions, highlighting the sensitivity of each system to formulation modifications. While emulsions stabilized by lecithin and saponin maintained stability over a period of 7 days, the addition of starch improved the stability of emulsions made with lecithin, especially when using a low surfactant concentration, particularly in terms of TSI. Despite the promising stability results, it is important to note that only two types of surfactants derived from plant sources were used. This limited quantity of sources does not offer a comprehensive enough representation of this field to contemplate the feasibility of replacing synthetic surfactants. However, this study not only contributes to understanding the complex interactions between surfactants and other components in food emulsions but also underscores the importance of carefully considering the choice of surfactant and the inclusion of additives to achieve desired properties. It is essential to thoroughly advance the study of the oxidation of emulsions, with this being a weakness in the present analysis. Future research directions will focus on studying the behavior and bioaccessibility of the nutrients present in O/W emulsions stabilized with ingredients of plant origin as they pass through the gastrointestinal tract of in vitro digestion systems. The objective is to verify the effectiveness of incorporating these ingredients into people’s diets, thus contributing to health and preventing diseases.

## Figures and Tables

**Table 1:** Oil droplet size and polydispersity index of the O/W emulsions stabilized by different plant-based surfactants.

Surfactant	Flaxseed Oil Concentration (% *w*/*w*)	Oil:Surfactant Ratio	d_10_ (µm)	d_50_ (µm)	d_90_ (µm)	PDI
Soy lecithin	25	5:1	0.14 ± 0.07 ^Aa^	0.66 ± 0.27 ^Aa^	2.19 ± 0.16 ^Aa^	3.11 ± 2.40 ^Aa^
		10:1	0.25 ± 0.05 ^Ab^	1.30 ± 0.49 ^Ab^	3.99 ± 0.57 ^Ab^	3.36 ± 1.96 ^Aa^
	30	5:1	0.10 ± 0.02 ^Aa^	0.40 ± 0.09 ^Aa^	1.30 ± 0.08 ^Aa^	3.09 ± 0.59 ^Aa^
		10:1	0.19 ± 0.02 ^Ab^	0.72 ± 0.14 ^Ab^	3.08 ± 0.03 ^Ab^	4.13 ± 0.91 ^Aa^
Quillaja saponin	25	5:1	0.03 ± 0.01 ^Ba^	0.11 ± 0.02 ^Ba^	0.31 ± 0.02 ^Ba^	2.41 ± 0.33 ^Ba^
		10:1	0.06 ± 0.01 ^Bb^	0.18 ± 0.01 ^Bb^	0.41 ± 0.02 ^Bb^	2.04 ± 0.14 ^Ba^
	30	5:1	0.08 ± 0.02 ^Ba^	0.19 ± 0.01 ^Ba^	0.37 ± 0.03 ^Ba^	1.53 ± 0.30 ^Ba^
		10:1	0.07 ± 0.00 ^Bb^	0.18 ± 0.01 ^Bb^	0.42 ± 0.02 ^Bb^	1.84 ± 0.16 ^Ba^

Mean values were significantly different with respect to surfactants (^A,B^) and oil:surfactant ratio (^a,b^) (Tukey’s post-hoc, *p* <0.05).

**Table 2:** Power law model parameters for the O/W emulsions stabilized by soy lecithin and Quillaja saponin.

Surfactant	Flaxseed Oil Concentration (% *w*/*w*)	Oil:Surfactant Ratio	Consistency Coefficient, K (Pa·s) (×10^−3^)	Flow Behavior Index, n (Dimensionless)	R^2^	RMSE
Soy lecithin	25	5:1	1.70 ± 0.00 ^Aa^	1.09 ± 0.12 ^a^	0.97	0.02
		10:1	0.54 ± 0.00 ^Ab^	1.41 ± 0.39 ^b^	0.93	0.11
	30	5:1	2.90 ± 0.00 ^Ba^	1.09 ± 0.11 ^a^	0.99	0.02
		10:1	0.19 ± 0.00 ^Bb^	1.57 ± 0.16 ^b^	0.98	0.02
Quillaja saponin	25	5:1	0.70 ± 0.00 ^Aa^	1.42 ± 0.31 ^a^	0.97	0.09
		10:1	0.20 ± 0.00 ^Ab^	1.57 ± 0.09 ^b^	0.98	0.02
	30	5:1	1.90 ± 0.00 ^Ba^	1.30 ± 0.06 ^a^	0.99	0.10
		10:1	2.00 ± 0.00 ^Bb^	1.21 ± 0.09 ^b^	0.99	0.02

Mean values were significantly different concerning oil percentage (^A,B^) and oil:surfactant ratio (^a,b^) (Tukey’s post-hoc, *p* < 0.05).

**Table 3:** Apparent viscosity of O/W emulsions stabilized by soy lecithin and Quillaja saponin with and without thickener.

Surfactant	Flaxseed Oil Concentration (% *w*/*w*)	Oil:Surfactant Ratio	Apparent Viscosity without Thickener (mPa·s)	Apparent Viscosity with Thickener (mPa·s)
Soy lecithin	25	5:1	1.89 ± 0.70 ^A^	691.0 ± 32.7 ^B^
		10:1	1.19 ± 0.18 ^A^	370.6 ± 6.1 ^B^
	30	5:1	4.36 ± 0.32 ^A^	1991.4 ± 164.6 ^B^
		10:1	2.50 ± 0.72 ^A^	759.4 ± 34.2 ^B^
Quillaja saponin	25	5:1	8.23 ± 0.63 ^A^	789.4 ± 16.3 ^B^
		10:1	3.90 ± 0.65 ^A^	679.7 ± 32.1 ^B^
	30	5:1	11.61 ± 2.14 ^A^	2117.3 ± 110.4 ^B^
		10:1	7.59 ± 0.30 ^A^	2133.2 ± 40.1 ^B^

Mean values were significantly different with respect to thickening addition (^A,B^) (Tukey’s post-hoc, *p* < 0.05).

## Data Availability

The original contributions presented in the study are included in the article/Supplementary Materials, further inquiries can be directed to the corresponding authors (Carolina Quezada and Elizabeth Troncoso). The data is designed to be used in other ongoing research and should be protected before official publication.

## Supplementary Materials

The following supporting information can be downloaded at https://www.mdpi.com/article/10.3390/foods13040513/s1, Figure S1: Time-dependent apparent viscosity of O/W emulsions sheared at 50 s^−1^ with and without thickener, stabilized by soy lecithin (A) and Quillaja saponin (B). The ap-parent viscosity axis is presented in a logarithmic scale; Figure S2. Evolution of delta backscattering (ΔBS) profiles (left) and a photographic record (right) of O/W emulsion stabilized by soy lecithin with and without thickener, monitored for 7 days; Figure S3. Evolution of delta backscattering (ΔBS) profiles (left) and a photographic record (right) of O/W emulsion stabilized by Quillaja saponin with and without thickener, monitored for 7 days.
