# Supplementary material for: Plant-Based Oil-in-Water Food Emulsions: Exploring the Influence of Different Formulations on Their Physicochemical Properties
Source: Foods. 2024 Feb 7;13(4):513. doi: 10.3390/foods13040513 (PMC10888144; doi:10.3390/foods13040513)
Supplement: Supplementary file 1 [file foods-13-00513-s001.zip › foods-2828842-supplementary.pdf]

Supplementary Material

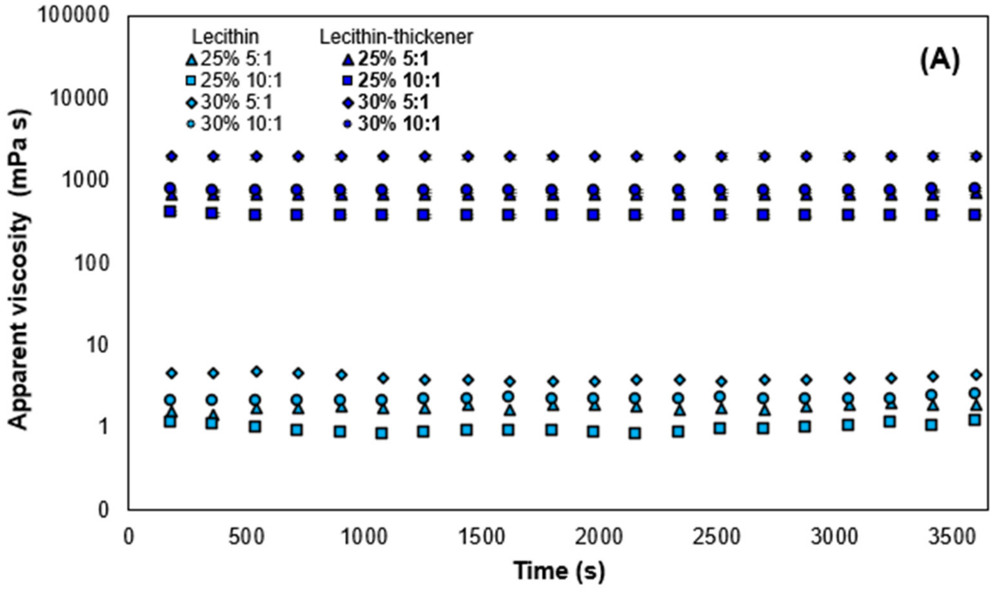

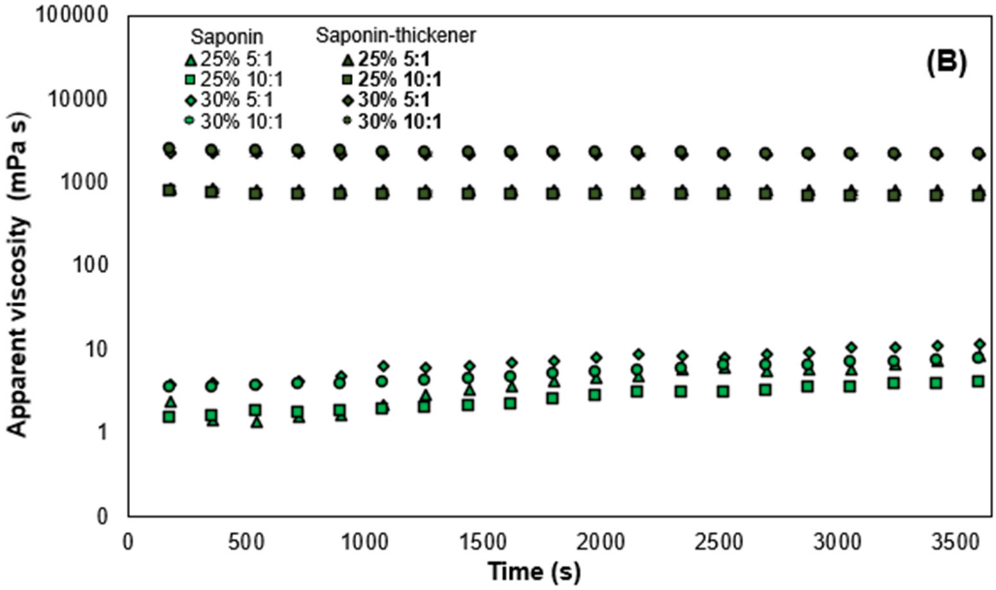

Figure S1. Time-dependent apparent viscosity of O/W emulsions sheared at 50 s^{-1} with and without thickener, stabilized by soy lecithin (A) Quillaja saponin (B). The apparent viscosity axis is presented in a logarithmic scale.

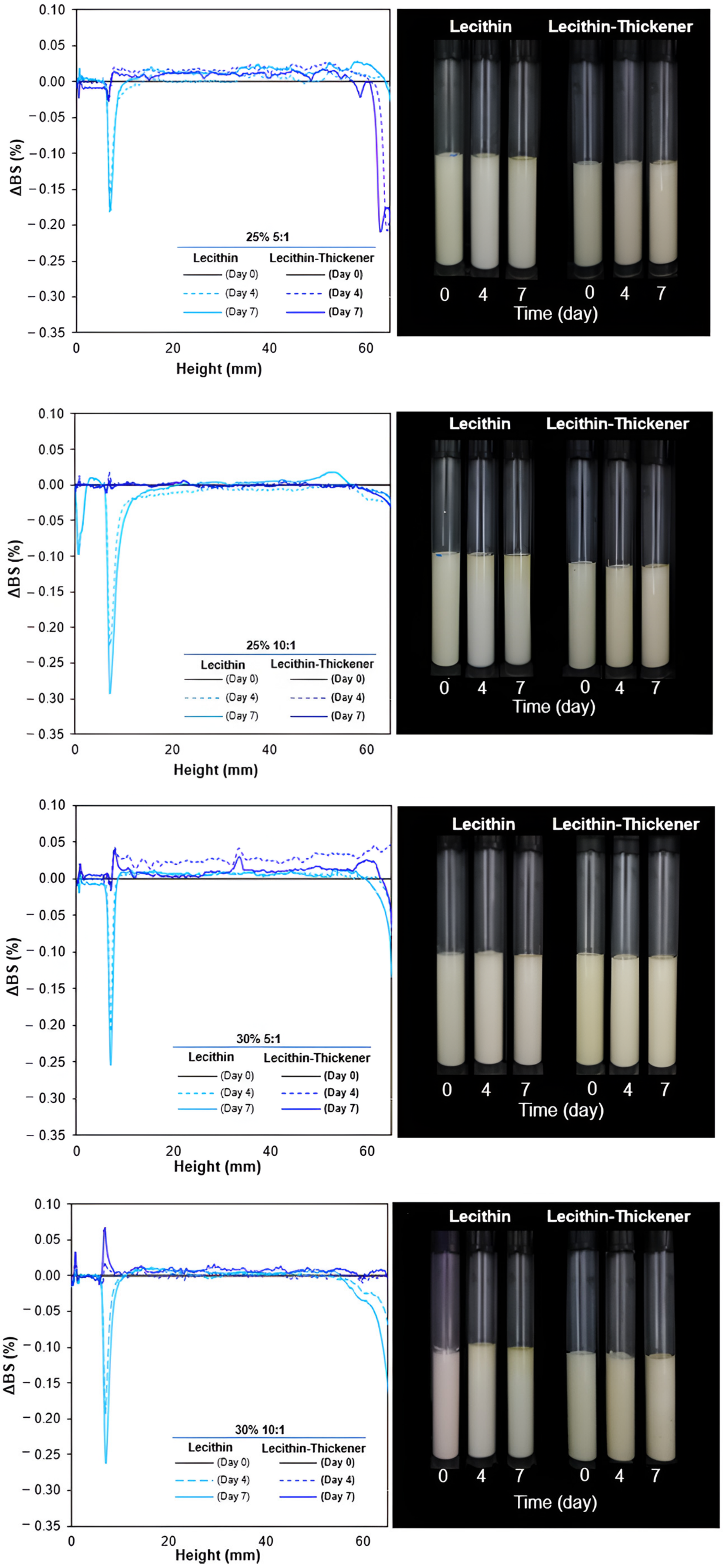

Figure S2. Evolution of delta backscattering (ΔBS) profiles (left) and a photographic record (right) of O/W emulsion stabilized by soy lecithin with and without thickener, monitored for 7 days.

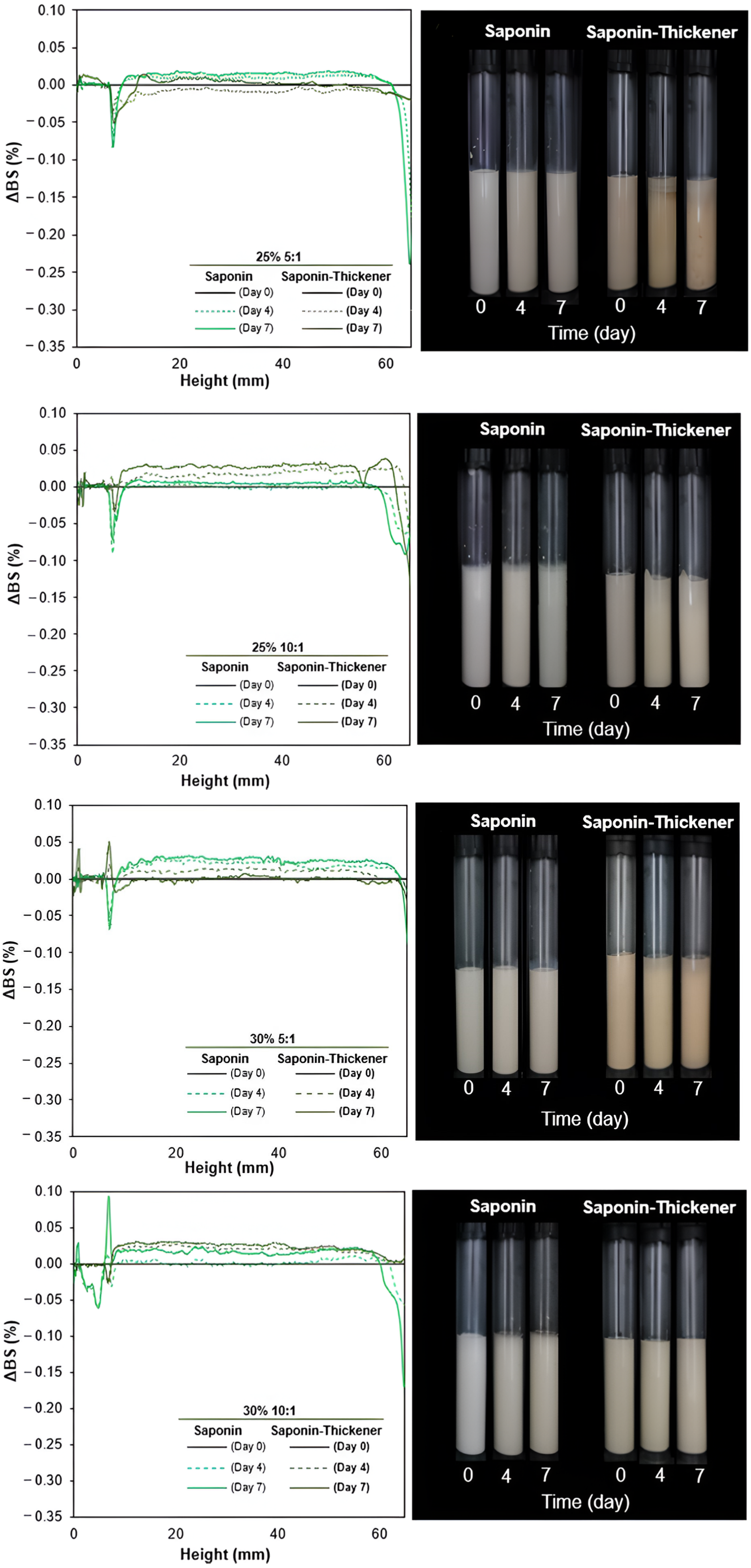

Figure S3. Evolution of delta backscattering (ΔBS) profiles (left) and a photographic record (right) of O/W emulsion stabilized by Quillaja saponin with and without thickener, monitored for 7 days.
